# Solubility of Cyclodextrins and Drug/Cyclodextrin Complexes

**DOI:** 10.3390/molecules23051161

**Published:** 2018-05-11

**Authors:** Phennapha Saokham, Chutimon Muankaew, Phatsawee Jansook, Thorsteinn Loftsson

**Affiliations:** 1 Faculty of Pharmacy, Rangsit University, Pathum Thani 12000, Thailand; phennapha.s@rsu.ac.th; 2 Faculty of Pharmacy, Siam University, 38 Petchkasem Road, Phasi Charoen District, Bangkok 10160, Thailand; chutimon.mua@siam.edu; 3 Faculty of Pharmaceutical Sciences, Chulalongkorn University, 254 Phyathai Road, Pathumwan, Bangkok 10330, Thailand; phatsawee.j@chula.ac.th; 4 Faculty of Pharmaceutical Sciences, University of Iceland, Hofsvallagata 53, 107 Reykjavik, Iceland

**Keywords:** cyclodextrin, complex, solubility, poorly soluble drug

## Abstract

Cyclodextrins (CDs), a group of oligosaccharides formed by glucose units bound together in a ring, show a promising ability to form complexes with drug molecules and improve their physicochemical properties without molecular modifications. The stoichiometry of drug/CD complexes is most frequently 1:1. However, natural CDs have a tendency to self-assemble and form aggregates in aqueous media. CD aggregation can limit their solubility. Through derivative formation, it is possible to enhance their solubility and complexation capacity, but this depends on the type of substituent and degree of substitution. Formation of water-soluble drug/CD complexes can increase drug permeation through biological membranes. To maximize drug permeation the amount of added CD into pharmaceutical preparation has to be optimized. However, solubility of CDs, especially that of natural CDs, is affected by the complex formation. The presence of pharmaceutical excipients, such as water-soluble polymers, preservatives, and surfactants, can influence the solubilizing abilities of CDs, but this depends on the excipients’ physicochemical properties. The competitive CD complexation of drugs and excipients has to be considered during formulation studies.

## 1. Introduction

Cyclodextrins (CDs) are cyclic oligosaccharides, formed by α-1,4-linked glucose units, with a hydrophilic outer surface and a lipophilic central cavity [1,2,3,4]. α-Cyclodextrin (αCD), β-cyclodextrin (βCD), and γ-cyclodextrin (γCD) are natural products that can be found in small amounts in various fermented consumer products, such as beer. Although the unsubstituted natural αCD, βCD, and γCD, and their complexes, are hydrophilic their solubility in aqueous solutions is somewhat limited, especially that of βCD. Consequently the more soluble βCD derivatives, such as 2-hydroxypropyl-βCD (HPβCD) and sulfobutylether βCD sodium salt (SBEβCD), are preferred for use in aqueous pharmaceutical solutions, such as parenteral drug formulations, even though both αCD and γCD can be found at low concentrations in parenteral formulations [5]. Monographs for αCD, βCD, and γCD and two βCD derivatives are in the European Pharmacopoeia and the United States Pharmacopeia/National Formulary (Table 1). CDs are included in over 40 marketed pharmaceutical products worldwide, in addition to numerous food, cosmetic, and toiletry products [2,6,7].

**Figure 1:**
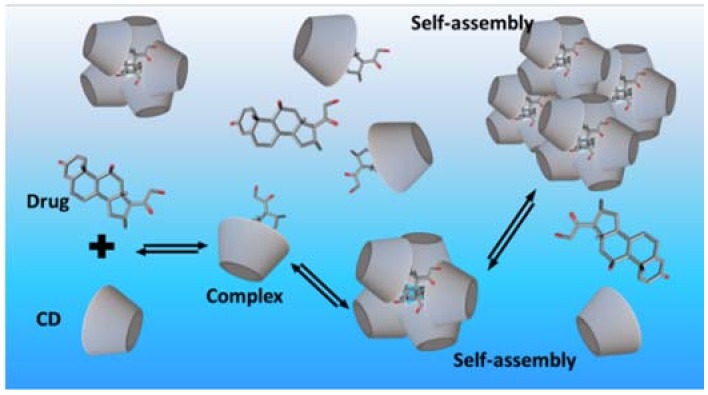
Formation of a cyclodextrin inclusion complex in an aqueous solution and self-assembly of cyclodextrin complexes.

Due to their ability to change physiochemical properties of drugs and other compounds, CDs are frequently referred to as enabling pharmaceutical excipients. CDs enable delivery of poorly water-soluble and chemically-unstable drugs to the body. Hence, CDs are able to convert biologically-active compounds that lack drug-like physiochemical properties into therapeutically-effective drugs. CDs (referred to as host molecules) are able to form inclusion complexes with drugs (referred to as guest molecules) by taking part of a drug molecule into the central CD cavity. This will change the physiochemical properties of the included drug. Formation of a drug/CD inclusion complex can, for example, increase the aqueous solubility of the drug, increase its chemical and physical stability, and enhance drug delivery through biological membranes. No covalent bonds are formed or broken during the complex formation and, in aqueous solutions, drug molecules bound within the CD inclusion complex are in dynamic equilibrium with free drug molecules (Figure 1) [8]. Drug molecules are readily released from the complex upon media dilution or by competitive complexation [9,10]. One or more drug molecules can form a complex with one CD molecule and one or more CD molecules can form a complex with one drug molecule. However, most commonly, one drug molecule (D) forms a complex with one CD molecule. The stoichiometry of the drug/CD complex (D/CD) is then 1:1 and the equilibrium constant (K_1:1_) defined as [11,12]:

(1) $$D+\mathrm{CD}\overset{K_{1\text{:}1}}{⇌}D/\mathrm{CD}$$

**Figure 2:**
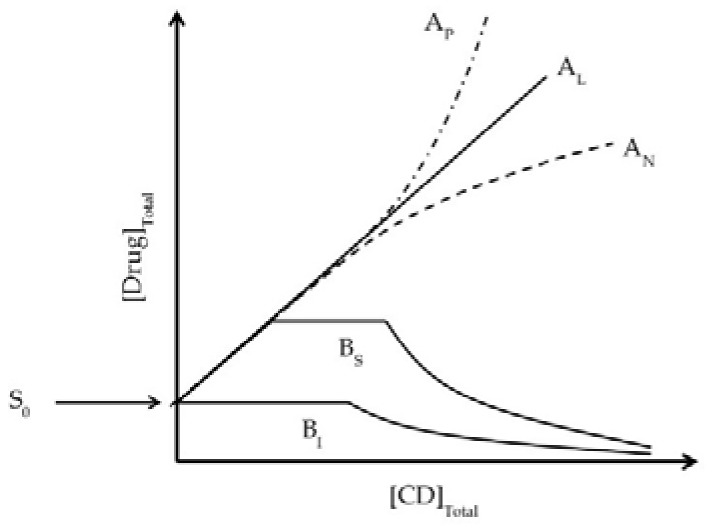
Types of phase-solubility diagrams according to Higuchi and Connors [11] showing how the total drug solubility changes with increasing CD concentration. A-type diagrams are formed when the drug/CD complex is soluble in the aqueous complexation media and they are usually associated with the water-soluble CD derivatives. B-type diagrams are observed when the complex has limited solubility in the media and these are usually associated with the natural CDs that have limited solubility in aqueous media. A_L_: linear diagram; A_P_: positive deviation from linearity; A_N_: negative deviation from linearity; B_S_: the complex has some but limited solubility; B_I_: the complex is insoluble.

The value of K_1:1_ can be calculated by Equation (2) where S_0_ is the intrinsic solubility of the drug (i.e., the solubility in the aqueous media when no CD is present), and Slope is the slope of the linear (i.e., A_L_-type) drug-CD phase solubility diagram (Figure 2):

(2) $$K_{1\text{:}1\;}=\frac{\mathrm{Slope}}{S_{0}\;\text{·}(1\;-\;\mathrm{Slope})}$$

However, the value of K_1:1_ is highly sensitive towards small changes in S_0_ and for poorly-soluble drugs it can be complicated to obtain accurate S_0_ values. Furthermore, self-association of lipophilic drug molecules in aqueous media can lead to erroneous results. Under such conditions, it can be more accurate to determine the complexation efficacy (CE):

(3) $$\mathrm{CE}\;={\;K}_{1\text{:}1}{\;\cdot \;S}_{0}\;=\frac{\mathrm{Slope}}{\left(1\;-\;\mathrm{Slope}\right)}$$

Drug/CD complexes, especially those of the natural CDs, have tendency to self-assemble in aqueous solutions to form aggregates (Figure 1). At elevated CD concentrations these aggregates can become large and precipitate as solid microparticles [1]. In addition, the natural CDs and their complexes have limited solubility in aqueous solutions. These solubility limitations can give rise to characteristic B-type phase-solubility diagrams displayed in Figure 2 [11].

The biopharmaceutical classification system (BCS) divides orally-administered drugs into four cases based on their solubility and intestinal permeability [13]. Drugs possessing favorable physiochemical properties are in Class I. They have adequate aqueous solubility and permeability to be well-absorbed from the gastrointestinal tract. In general, they gave good oral bioavailability. Drugs in Class II have inadequate aqueous solubility, but good permeability when in solution. Thus, their absorption from the gastrointestinal tract is slow and dissolution dependent. Drugs in Class III have adequate aqueous solubility but poor permeability, most often due to their very hydrophilic nature. Frequently such drugs are administered via parenteral injection. Finally, drugs in Class IV have both low aqueous solubility and are poorly absorbed from the gastrointestinal tract. Thus, they have very poor oral bioavailability and can be difficult to formulate as parenteral solutions. CDs can improve oral bioavailability of Class II drugs and sometimes also of Class IV drugs while they have negligible effect on Class III drugs and can under certain conditions even hamper absorption of some Class I drugs [10]. The BCS can be adapted to other types of dosage forms intended for non-oral administration [14]. Here the aqueous solubility of CDs and their complexes is reviewed, as well as the effect of CD concentrations on drug permeation through biological membranes.

## 2. Physiochemical Properties of Cyclodextrins

The secondary hydroxyl groups on the CD molecule are located on the wider rim of the molecule and the primary hydroxyl groups on the narrow rim make them hydrophilic [12,15]. Due to their hydrophilic outer surface and their large number of hydrogen bond donors and acceptors, CDs have very negative Log*P_o_*_/*w*_ value (i.e., the logarithmic value of the octanol/water partition coefficient) (Table 2) [4,16]. In aqueous solutions, CDs are susceptible to acid hydrolysis at low pH resulting in ring opening and formation of various linear oligosaccharides and glucose units, but they are stable under alkaline conditions. The hydroxyl groups attached to the rim start to deprotonate at pH about 12 [17,18]. Depending on the determination method and the location of the hydroxyl groups, the pKa values of the natural CDs have been reported to be between 12.1 and 13.5 [19]. The main difference of the three natural CDs, besides the size of their central cavity, is their aqueous solubility (Table 2). βCD is the least soluble but, at the same time, possesses the most suitable cavity size for complex formation with many drugs [20,21]. The poor solubility can be explained in term of molecular rigidity of CD molecule, and the effect caused by the intermolecular hydrogen bonding in the crystal state [22,23]. Particularly, the hydrogen bond formation between neighboring C2-OH and C3-OH in the βCD molecule leads to the so-called complete secondary belt resulting in inflexible structure and reduced ability of the βCD molecule to form intermolecular hydrogen bond with surrounding water molecules [15]. Molecular dynamic simulations have shown high water density and strong ordering of water molecules around the βCD molecule [24,25]. This indicates that water molecules surrounding dissolved βCD molecules have unfavorable enthalpy and low entropy, which can explain the low water solubility of βCD compared to other natural CDs. In contrast, αCD has incomplete belt of hydrogen bonds and γCD has non-coplanar structure. Consequently, both αCD and γCD possess higher solubility in water.

To enhance solubilizing potential of the natural CDs, including their complexation capacity, CD derivatives have been prepared by reacting the secondary and/or primary OH groups of the molecule with a wide variety of substituents [26,27]. Randomly methylated and hydroxypropylated CDs and sulfobutylether CDs are manufactured on an industrial scale and often used in pharmaceutical products, whereas other CD derivatives are utilized for specific purposes, such as the removal of pollutants from the environment and reagents for drug analysis [28,29]. Attachment of these substituents disrupts the regular hydrogen bonding network within native CD molecule increasing their ability to interact with the surrounding water molecules [30]. The result can be over 100-fold increase in their aqueous solubility [31,32,33,34]. For example, the sulfobutylether βCD anionic alkyl chains form an extremely hydrophilic exterior surface. These anionic chains provide for electrostatic repulsion resulting in extension of the hydrophobic central cavity and thereby intensifying its solubilizing potential [35]. Most frequently, modified natural CDs exist in amorphous isomeric mixtures of various degree of substitution (DS). The DS has a great influence on the physicochemical properties of CDs and their ability to form complexes. For instance, randomly-methylated βCD (RMβCD) has the highest solubility in water when the degree of substitution is about 14, that is, when two-thirds of the OH-groups have been replaced by methoxy groups [36]. Increasing the DS above 14 decreases the RMβCD solubility. The cavity diameter of CD derivatives is similar to their parent CDs. However, an effect of substituent location on the cavity volume has been observed [37]. It has been observed that the hydroxypropylation of OH groups at the O-2 position results in a more spread out configuration, whereas the substitution of OH groups at the O-6 position reduces the water density inside the CD cavity [30,38,39]. Unlike native CDs that have negligible surface activity, some CD derivatives manifest such behavior. It is reported that methylated and hydroxyalkylated CD molecules reduce the surface tension of water [40,41]. The increase in surface activity is proportional to the increased degree of substitution. On the other hand, derivatives with polar ionic groups, such as carboxylate ion and sulfobutyl groups, have no influence on surface activity [35]. Table 2 lists dimensional structures and physicochemical properties of the three most common natural CDs and some of their more common derivatives.

## 3. How Much Solubilization Is Needed?

**Figure 3:**
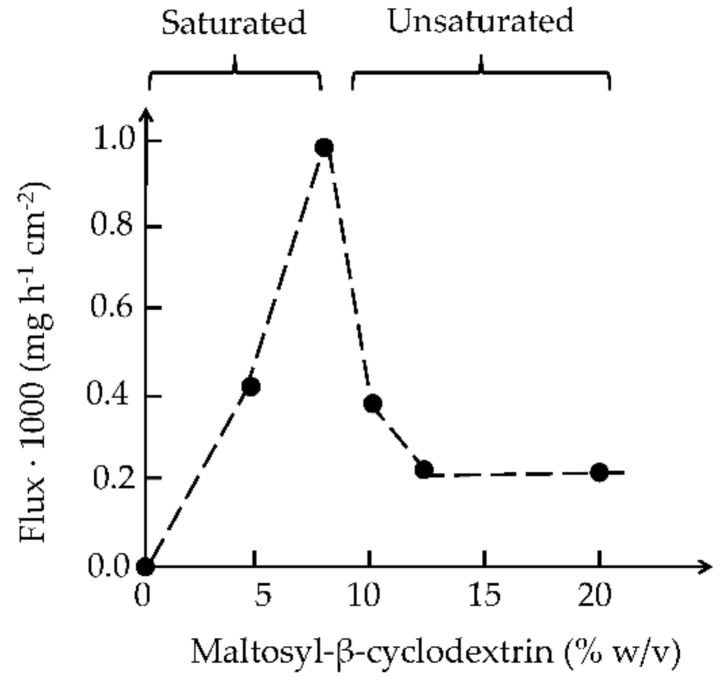
The effect of maltosyl-β-cyclodextrin concentration on the permeation of hydrocortisone through hairless mouse skin in vitro. The amount of hydrocortisone in the aqueous donor medium was kept constant 16 mg/mL but the maltosyl-β-cyclodextrin concentration was from 0 to 20% (*w*/*v*). Below 8% (*w*/*v*) maltosyl-β-cyclodextrin the medium was hydrocortisone suspension in water, but hydrocortisone solution in water at higher concentrations. Based on unpublished results.

CDs can both enhance and hamper drug permeation through biological membranes. Although active drug transport through biological membranes does exist, drug molecules are mainly transported via passive diffusion through the membranes. In general, the driving force for passive diffusion through an aqueous environment (e.g., mucus) into and through membranes, such as mucosa is not the concentration gradient but the gradient of chemical potential, which is a continuous function across interfaces [50]. Likewise, the partitioning of drug molecules from a membrane exterior into the outermost membrane layer is controlled by the chemical potential. Thus, maximum drug permeation from an aqueous exterior into and through biological membrane is expected to be obtained when the drug is at its highest thermodynamic potential [50]. In other words, maximum drug permeation is obtained when the aqueous membrane exterior is saturated with the drug [51]. However, the amount of drug permeating through a membrane also depends on the concentration of dissolved drug at the membrane exterior. Figure 3 shows the flux of hydrocortisone through hairless mouse skin in vitro. The skin was mounted in a Franz diffusion cell where the donor phase was unstirred (i.e., consisted of an unstirred diffusion layer), but the receptor phase was stirred. The total amount of hydrocortisone (i.e., dissolved and undissolved) in the donor phase was kept constant at 16 mg/mL while the CD concentration was increased from 0 to 20% (*w*/*v*). About 8% (*w*/*v*) CD was needed to solubilize 16 mg of hydrocortisone in 1 mL of the donor phase (i.e., the aqueous medium). Thus, increasing the CD concentration from 0 to 8% (*w*/*v*) increases the amount of dissolved drug in the donor phase. At these CD concentrations, the donor phase was always saturated with the drug and, thus, the drug is always at its maximum thermodynamic potential. Under these conditions, the drug molecules have maximum tendency to leave the donor phase and partition into the skin. However, only dissolved drug molecules can partition into the skin and, thus, increasing the concentration of dissolved drug molecules through formation of water-soluble drug/CD complexes increases the number of drug molecules that are able to partition into the skin and then permeate through the skin into the receptor phase. Increasing the CD concentration beyond 8% (*w*/*v*) decreases the thermodynamic potential of the drug. The solubility of hydrocortisone in 10% (*w*/*v*) CD solution is about 20 mg/mL and about 26 mg/mL at 13% (*w*/*v*) CD. The donor phase was no longer saturated with the drug and the drug molecules have decreased tendency to leave the donor phase and partition into the membrane. To ensure maximum drug permeation through biological membranes one should only add just enough CD to the pharmaceutical formulation to solubilize the entire drug dose. Too little or too much CD will result in less than optimum drug flux through the membrane. However, small excess CD has to be included in aqueous drug solutions to prevent drug precipitation during storage and handling.

In solid dosage forms such as tablets adequate amount of CD should be included to prevent dissolution controlled drug absorption from the gastrointestinal tract [10]. Excess CD can hamper absorption from the gastrointestinal tract and, for example, αCD is used to bind and prevent absorption of dietary fat [52]. The unsubstituted natural αCD, βCD, and γCD frequently form drug/CD complexes that have limited solubility in water. However, their solubility is most often sufficient to prevent dissolution limited absorption, and since natural CDs have lower molecular weight than their more water-soluble derivatives their formulation bulk will be lower. General observations regarding the amount of CD to be included in pharmaceutical formulations are listed in Table 3.

## 4. The Effect of the Guest Molecule on the Cyclodextrin Solubility

Not only are aqueous solubilities of drugs affected by the formation of drug/CD complexes, but also that of the CDs themselves. According to the phase-solubility diagram classification system that was introduced by Higuchi and Connors [11], linear A_L_-type diagrams show that the total drug solubility increases as a function of CD concentration through formation of soluble drug/CD complexes. If one molecule of drug forms a complex with one molecule of CD, the slope of a straight line is less than unity and the value of K_1__:__1_ can be calculated by applying Equation (2). A_P_- or A_N_-type phase-solubility diagrams (i.e., displaying positive or negative deviation from linearity, respectively) suggest formations of higher-order drug/CD complexes [20,53]. If the slope of a linear diagram is greater than unity, but less than 2, the complex formed is likely to be of second, or higher, order with respect to the drug, but first-order with respect to CD. For example, a K_2__:__1_ value of drug/CD complex can be determined by [46]:

(4) $$K_{2:1}\;=\;\frac{\mathrm{slope}}{\left(2-\mathrm{slope}\right)S_{0}^{2}}$$

**Figure 4:**
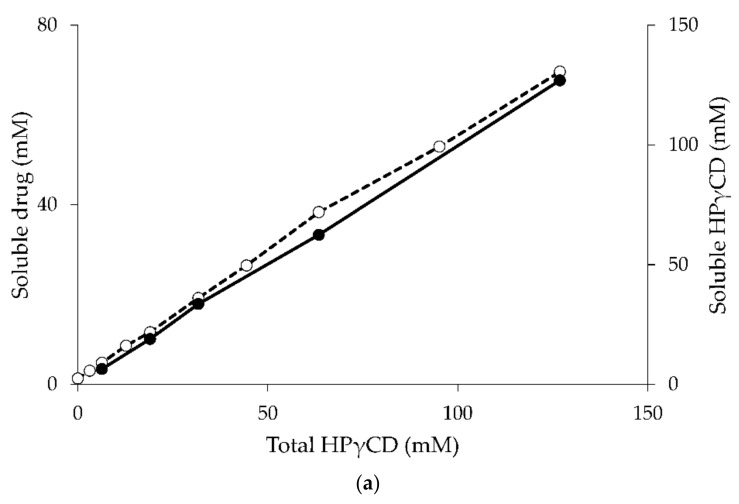

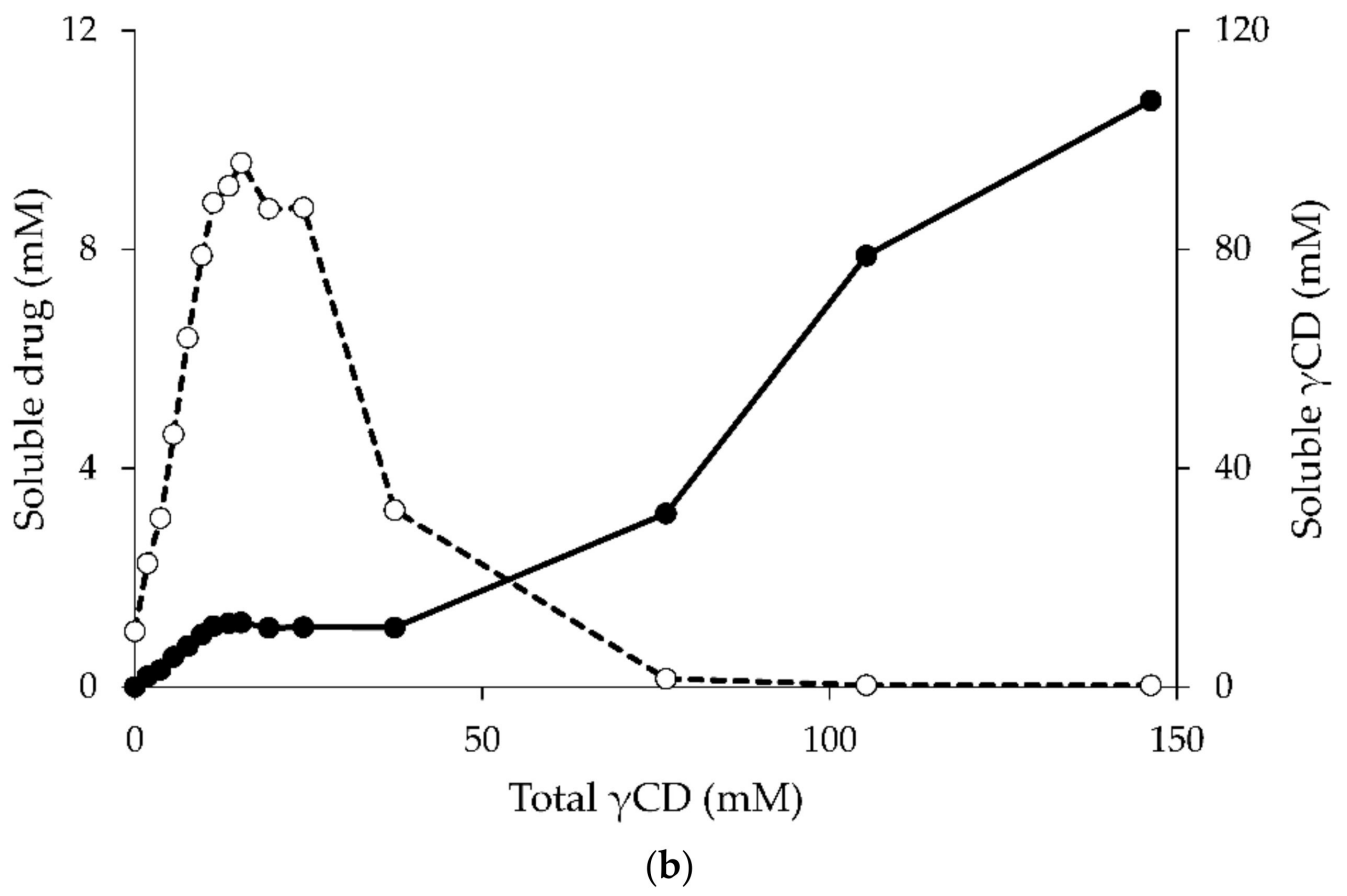
The phase-solubility diagram of hydrocortisone/HPγCD (a) [58] and hydrocortisone/γCD (b) [59] complex. Concentrations of the soluble drug (open circle) and CD (filled circle) are plotted against the concentration of total CD.

The A_P_-type phase-solubility diagram suggests the formation of higher-order complexes with respect to CD (e.g., formation of 1:2 drug/CD complex). The complex stoichiometry and equilibrium constant (K_2__:__1_) can then be determined by fitting the solubility results to a quadratic model [20,54]. The tendency of a given drug and CD to form a complex is expressed by a stability constant for the complex (K_m__:__n_), where m and n are the number of molecules of the drug and CD forming the complex, respectively, or the equilibrium binding constant (K_a_) defined by the ratio of association (k_a_) and dissociation (k_d_) rate constants [55,56]. The K_a_ values of most drug/CD complexes are less than 10^5^ M^−1^, indicating that the drug and CD interactions are relatively weak [20,56]. Moreover the forward (k_a_) and reverse (k_d_) reactions are very fast and the relaxation time is short (less than 1 s) [55,57], indicating that drug and CD molecules in complex are in rapid equilibrium with free molecules in the solution. Thus, in aqueous solutions drug/CD complexes are in dynamic equilibrium with free drug and CD molecules. Studies of dissolved γCD in aqueous complexation media of indomethacin/γCD, diclofenac sodium/γCD, and amphotericin B/γCD (all of which display A_L_-type phase-solubility diagrams) show that the determined concentrations of γCD are almost identical to the initial concentration of dissolved γCD before the addition of the drug [58]. The influence of drug concentration on CD solubility in complexation media is negligible when the phase-solubility diagram of the drug/CD complex presents as A-type (Figure 4a).

In general, the water-soluble CD derivatives form A-type phase-solubility diagrams, whereas the B-type diagrams are mainly observed when the natural CDs form complexes with poorly-soluble drugs [20]. The B-type phase-solubility diagrams indicate the formation of complexes with limited solubility in the complexation media. The initial linear region of B_s_-type diagrams can be regarded as A_L_-type diagrams. In this region of the diagrams drug molecules do not affect the CD solubility. In the plateau region of the B-type diagram the drug solubility is constant even when the CD concentration is increased, indicating the formation of drug/CD complexes with limited solubility and that the CD solubility is depressed by the presence of the drug [58,59]. The amount of dissolved CD and drug in aqueous complexation media are constant through the length of plateau region (Figure 4b) [59,60]. However, the amounts of drug and CD in the precipitate can differ. Schönbeck reports that precipitate obtained from phase-solubility studies consists of solid drug (i.e., hydrocortisone in excess) and precipitated drug/γCD complex. The ratio of γCD to drug in precipitate increases as a function of the γCD concentration (i.e., the amount of precipitated drug decreases, whereas the amount of precipitated drug/γCD complex increases) indicating that the stoichiometry of the drug/CD inclusion complex gradually changes from 1:1 to higher-order drug/CD complexes [61]. Since the solubility of the drug/CD complex is limited, higher-order drug/CD complexes precipitate resulting in decreased CD solubility in the aqueous complexation media. The descendent region of B-type phase-solubility diagrams show that solubility of the drug decreases when the concentration of CD increases, indicating that CD preferably forms self-assembled aggregates and the solubility of CD gradually increases even in the presence of the drug [60]. The absence of solid pure drug in the precipitate from phase-solubility studies also indicates that only higher-order drug/CD complexes are being formed [61]. In conclusion, the CD solubility, especially that of the natural CDs, can be decreased in the presence of drugs if high-order drug/CD complexes, for example 1:2 or 2:1, are formed and then precipitated from the media.

## 5. Excipients and Cyclodextrin Solubility

In pharmaceutical products, not only drugs and CDs are present as drug/CD complexes, but also various excipients, such as antioxidants, antimicrobial agents, surfactants and polymers. These excipients can enhance or hamper the CD solubilization of drugs, as shown in Table 4. Preservatives, such as propyl- and methylparaben, can compete with drug molecules and expel them from the CD cavities and, thus, reduce CD solubilization of the drugs [62,63]. In addition, CD complexation of the preservatives can reduce their antimicrobial efficacy. Therefore, formulation scientists need to adjust the amount of preservative in CD-containing formulations to obtain the desired preservative efficacy and safety [64]. The additive or synergistic effects of excipients on the drug solubility through CD inclusion complexes have been reviewed [20,65]. Various additives that are commonly used in pharmaceutical formulations, such as organic acids or bases, organic salts (counterions), cosolvents, metal ions, and water-soluble polymers, can increase the complexation efficacy (CE) of CDs via stabilization and solubilization of drug/CD nanoparticles. Recently, we have shown that CDs and drug/CD complexes can self-assemble and form complex aggregates in aqueous solutions, which can enhance the drug solubility [54,66,67]. Water-soluble polymers play an important role in the stabilization of aggregates. Formation of ternary drug:CD:polymer complexes can be promoted by heating the media, for example, in an autoclave (121 °C for 15–20 min) or in an ultrasonic bath (e.g., 60–70 °C for 1 h) [68]. It has been shown that the addition of small amounts of water-soluble polymer reduces the formulation bulk, decreasing the manufacturing cost and increasing the possibility of using CDs as solubilizers in various solid dosage forms. For instance, the amount of βCD or SBEβCD required to solubilize 3.0 mg of glibenclamide were 300 mg and 1200 mg, respectively. Addition of hydroxypropyl methylcellulose (HPMC) to the binary complexes reduced the amount of CD in the formulation to 120 mg and 250 mg, respectively [69].

The excipients in the pharmaceutical formulations containing CD may increase or decrease the ability of CD to solubilize drugs depending on their nature and physicochemical properties. Thus, the exact amount of CD needed in a given formulation should be determined by studies (e.g., phase-solubility studies) in a medium which composition is close to that of the final formulation. The competitive complexation effect of the secondary drug should also be considered [70].

## 6. Conclusions and Directions

Various physiochemical properties of drugs can be altered through CD complexation, especially drug solubility in aqueous biological media. In aqueous media, drug molecules of appropriate size and structure will enter into the central cavity of CD molecules to form water-soluble complexes and, frequently, enhanced total drug solubility is observed. A_L_-type phase solubility diagrams represent linear relationships between concentrations of dissolved drug and amounts of CD added to an aqueous medium. However, when B-type diagrams are observed, the CD molecules and their complexes self-assemble to form aggregates that possess limited solubility. While drug/CD complexes are in dynamic equilibrium with free drug and CD molecules in aqueous media, CD aggregates frequently precipitate from the media. The solid CD aggregates decrease CD solubility and this will, again, influence formation of drug/CD complexes. Pharmaceutical excipients, for example water-soluble polymers, are able to hamper this type of CD precipitation via formation of ternary complexes leading to enhanced CD complexation efficacy. Since CD forms complexes with wide variety of guest molecules, including drugs and pharmaceutical excipients, competitive complexation should always be take into account. Therefore, the amount of CD and the type and composition of pharmaceutical excipients used in pharmaceutical formulation needs to be carefully selected.

## Figures and Tables

**Table 1:**
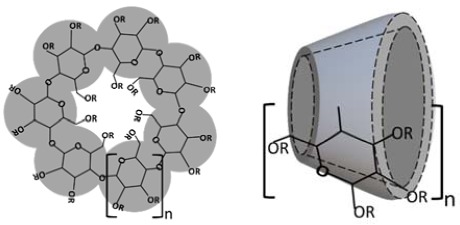
Cyclodextrins with pharmacopoeia monographs.

Cyclodextrin	n	R = H or	Abbreviation	Synonyms	Pharmacopoeia Monographs ^1^
α-Cyclodextrin	0		αCD	alfadex	Ph.Eur., USP-NF, JPC
β-Cyclodextrin	1		βCD	betadex	Ph.Eur., USP-NF, JPC
2-Hydroxypropyl-β-cyclodextrin	1	-CH_2_CHOHCH_3_	HPβCD	hydroxypropylbetadex	Ph.Eur., USP-NF
Sulfobutylether β-cyclodextrin sodium	1	-(CH_2_)_4_SO_3_^−^ Na^+^	SBEβCD	betadex sulfobutyl ether sodium	USP-NF
γ-Cyclodextrin	2		γCD	gammadex	Ph.Eur., USP-NF, JPC

^1^ The European Pharmacopoeia (Ph.Eur.), the United States Pharmacopeia and the National Formulary (USP-NF), and the Japanese Pharmaceutical Codex (JPC).

**Table 2:** The cavity size and some important physicochemical properties of natural CDs and some of their derivatives.

Types	Substituent	^1^ DS	Inner Cavity Diameter (Å)	Hydrogen Donors	Hydrogen Acceptors	Solubility (mg/mL, 25 °C)	Log P*_o_*_/*w*_	Surface Tension (mN/m)	References
**Naural CD**									
αCD	H	0	4.7–5.3	18	30	145	−13	71	[1,42]
βCD	H	0	6.0–6.5	21	35	18.5	−14	71	[1]
γCD	H	0	7.5–8.3	24	40	232	−17	71	[1]
**Modified CD**									
HPαCD	-CH_2_-CHOH-CH_3_	3.6	4.5–5.3	18	36	-	-	-	[43]
CMβCD	-CH_2_-CO_2_H	3–5	-	21	49	50	−4.9	-	[32]
DMβCD	-CH_3_	12–16	5.8–6.5	7	35	570	-	62	[34]
RMβCD	-CH_3_	9.7–13.6	-	9	35	>500	−6	57.5–54.1	[1,44,45]
TMβCD	-CH_3_	21	4–7	0	35	310	-	56	[34,46]
HEβCD	-CH_2_-CH_2_OH	3.6	-	21	42	>2000	-	-	[26,47]
HPβCD	-CH_2_-CHOH-CH_3_	2.8–10.5	6.0	25	39	>1200	−11	54.8–57.5	[1,47]
SBEβCD	(CH_2_)_4_-SO_3_Na	6.2–6.9	-	21	35	>1200	<−10	71	[1]
HPγCD	-CH_2_-CHOH-CH_3_	3.0–5.4	8.0	24	45	800	−13	-	[1]
SBEγCD	(CH_2_)_4_-SO_3_Na	4–8	-	-	-	-	-	-	[43,48]
SUG	-SCH_2_CH_2_CO_2_Na	8	7.5–8.3	24	48	Very soluble	−16	72.2	[1]
**Branched CD**									
G_1_βCD	glucosyl	1	6.0–6.5	24	40	970	−9	71	[26,49]
G_2_βCD	maltosyl	1	-	27	45	>1500	−9	72	[33]
GUGβCD	glucoronylglucosyl	1	-	-	-	>2000	-	73	[33]

^1^ DS is defined as the average number of substituents per one CD molecule; DMαCD, dimethyl-αCD; TMαCD, trimethyl-αCD; HPαCD, 2-hydroxypropyl-αCD; CMβCD, carboxymethyl-βCD; DMβCD, dimethyl-βCD; TMβCD, trimethyl-βCD; HEβCD, hydroxyethyl-βCD; DMγCD, dimethyl-γCD; TMγCD, trimethyl-γCD; HPγCD, hydroxypropyl-γCD, SBEγCD, sulfobutylether-γCD sodium salt; SUG, sugammadex; G_1_βCD, glucosyl-βCD; G_2_βCD, maltosyl-βCD; GUGβCD, glucoronyl-glucosyl-βCD.

**Table 3:** In general, pharmaceutical formulations should contain sufficient CD to solubilize the entire drug dose. However, how much solubilization is needed will depend on the formulation.

Formulation	Amount of CD	Comments
Parenteral solutions	Sufficient to solubilize the drug dose in, for example, 10 mL of water.	Significant excess CD (as much as two to three times what is needed to solubilize the drug) is frequently included in parenteral formulations, especially those that are given intravenously. This will not affect the drug pharmacokinetics since the drug is almost instantaneously released from the complex upon dilution in the blood circulation.
Solid oral dosage forms (e.g., tablets and capsules)	Sufficient to increase aqueous solubility the drug dose to prevent dissolution controlled absorption.	The formulation bulk usually limits the amount of CD that can be included in solid dosage forms. For example, if βCD (molecular weight 1135 Da) is used in a solid dosage form containing 100 mg of a drug with molecular weight 250 Da the formulation bulk will be increased by over five-fold.
Oral solutions	Sufficient to dissolve the drug dose in the aqueous vehicle.	Excess of CD (e.g., ≥20) should be used to prevent drug precipitation upon storage and usage of the formulation. Due to formulation dilution in the gastrointestinal tract some excess CD will not hamper the drug release. However, large excess (over 50 to 100) can hamper the drug release.
Topical solutions with limited dilution upon administration (e.g., eye drops)	Sufficient to dissolve the drug dose in the aqueous vehicle.	Only a small excess of CD (e.g., 10 to 20%) should be used to prevent drug precipitation upon storage and usage of the formulation. Excess amounts of CD (e.g., more than 10%) can reduce topical bioavailability of the drug.

**Table 4:** Effects of some pharmaceutical excipients on the cyclodextrin solubilization.

Excipients	Examples	Effect on Complexation Enhancement	Some Brief Observations	References
Acids, bases, inorganic/organic salts	hydrochloride, citrate, aspartate, mesylate, maleate, tartrate, phosphate, acetate	Increase intrinsic solubility of drugs (S_0_) and/or the apparent stability constant (K_1:1_) resulting in increased CE	Salt formation of ziprasidone mesylate enhance the CE of drug up to 100 and 240 times in aqueous HPβCD and SBEβCD solutions when compared with the free base of drug	[71]
			Ternary complex of terfenadine/βCD/inorganic acid (phosphate, citrate) induce the synergistic effect of CD solubilization	[72]
			The addition of sodium acetate into the complexing medium containing βCD could increase hydrocortisone solubility up to 220%	[73]
		Enhance S_0_ but in most cases decrease K_1:1_	K_1:1_ of fluasterone/HPβCD complex decreases with increasing ethanol concentration but the drug solubility increased at high ethanol concentration (>40% *v*/*v*)	[74]
Cosolvents	ethanol, propylene glycol (PG)		Ternary complex of diazepam/PG/βCD increased the diazepam solubility than that of the mixture of PG and water	[75]
		Hamper complexation by the competitive effect	At higher concentrations of PG, the methyltestosterone solubility in presence of HPβCD decreased possibly due to the complex dissociation	[76]
Water-soluble polymers	HPMC, Na CMC, PVA, PVP	Formation of ternary complex (drug/CD/polymer) that can increase K_1:1_	Polymers and CDs can form water-soluble complexes with poorly water-soluble drugs, for example, acetazolamide, carbamazepine hydrocortisone, naproxen, pregnenolone, tropicamide, etc. have been reviewedSynergistic solubilization effect is possible through micellar-like solubilization or stabilization of self-assembled CD and/or CD complex aggregates	[20,77,78,79]
Metal ions	Mg^2+^	Enhance CE by increasing S_0_ via formation of drug/CD/metal ion complexes	Synergistic solubilization of quinolone was obtained when the addition of Mg^2+^ to the drug/HPβCD complexes	[80]

Hydroxypropyl methylcellulose (HPMC); sodium carboxymethylcellulose (Na CMC); polyvinyl alcohol (PVA), polyvinyl pyrrolidone (PVP).

## Abbreviations

The following abbreviations are used in this manuscript:

CD	Cyclodextrin
CE	Complexation efficiency
CMβCD	Carboxymethyl-βCD
DMβCD	Dimethyl-βCD
DMαCD	Dimethyl-αCD
DMγCD	Dimethyl-γCD
DS	Degree of substitution
G1βCD	Glucosyl-βCD
G2βCD	Maltosyl-βCD
GUGβCD	Glucoronyl-glucosyl-βCD
HEβCD	Hydroxyethyl-βCD
HPMC	Hydroxypropyl methylcellulose
HPαCD	2-hydroxypropyl-αCD
HPγCD	Hydroxypropyl-γCD
Na CMC	Sodium carboxymethylcellulose
PVA	Polyvinyl alcohol
PVP	Polyvinyl pyrrolidone
SBEγCD	Sulfobutylether-γCD
SUG	Sugammadex
TMβCD	Trimethyl-βCD
TMαCD	Trimethyl-αCD
TMγCD	Trimethyl-γCD
αCD	α-Cyclodextrin
βCD	β-Cyclodextrin
γCD	γ-Cyclodextrin

## References

[1] Jansook P., Ogawa N., Loftsson T. (2018). Cyclodextrins: Structure, physicochemical properties and pharmaceutical applications. Int. J. Pharm..

[2] Astray G., Gonzalez-Barreiro C., Mejuto J.C., Rial-Otero R., Simal-Gándara J. (2009). A review on the use of cyclodextrins in foods. Food Hydrocoll..

[3] Muankaew C., Loftsson T. (2018). Cyclodextrin-based formulations: A non-invasive platform for targeted drug delivery. Basic Clin. Pharmacol. Toxicol..

[4] Kurkov S.V., Loftsson T. (2013). Cyclodextrins. Int. J. Pharm..

[5] FDA U.S.F.D.U.S. Inactive Ingredient Search for Approved Drug Prodructs. https://www.accessdata.fda.gov/scripts/cder/iig/getiigWEB.cfm.

[6] Arima H., Motoyama K., Higashi T. (2017). Potential use of cyclodextrins as drug carriers and active pharmaceutical ingredients. Chem. Pharm. Bull..

[7] Hu Q.-D., Tang G.-P., Chu P.K. (2014). Cyclodextrin-based host–guest supramolecular nanoparticles for delivery: From design to applications. Acc. Chem. Res..

[8] Stella V.J., Rao V.M., Zannou E.A., Zia V. (1999). Mechanisms of drug release from cyclodextrin complexes. Adv. Drug Del. Rev..

[9] Kurkov S.V., Madden D.E., Carr D., Loftsson T. (2012). The effect of parenterally administered cyclodextrins on the pharmacokinetics of coadministered drugs. J. Pharm. Sci..

[10] Loftsson T., Moya-Ortega M.D., Alvarez-Lorenzo C., Concheiro A. (2016). Pharmacokinetics of cyclodextrins and drugs after oral and parenteral administration of drug/cyclodextrin complexes. J. Pharm. Pharmacol..

[11] Higuchi T., Connors K.A. (1965). Phase-solubility techniques. Adv. Anal. Chem. Instrum..

[12] Brewster M.E., Loftsson T. (2007). Cyclodextrins as pharmaceutical solubilizers. Adv. Drug Del. Rev..

[13] Amidon G.L., Lennernas H., Shah V.P., Crison J.R. (1995). A theoretical basis for a biopharmaceutic drug classification: The correlation of in vitro drug product dissolution and in vivo bioavailability. Pharm. Res..

[14] Loftsson T. (2002). Cyclodextrins and the biopharmaceutics classification system of drugs. J. Incl. Phenom. Macrocycl. Chem..

[15] Szejtli J. (1998). Introduction and general overview of cyclodextrin chemistry. Chem. Rev..

[16] Loftsson T., Vogensen S.B., Brewster M.E., Konráðsdóttir F. (2007). Effects of cyclodextrins on drug delivery through biological membranes. J. Pharm. Sci..

[17] Kondo H., Nakatani H., Hiromi K. (1990). In vitro action of human and porcine α-amylases on cyclomalto-oligosaccharides. Carbohydr. Res..

[18] Lumholdt L.R., Holm R., Jørgensen E.B., Larsen K.L. (2012). In vitro investigations of α-amylase mediated hydrolysis of cyclodextrins in the presence of ibuprofen, flurbiprofen, or benzo[a]pyrene. Carbohydr. Res..

[19] Gaidamauskas E., Norkus E., Butkus E., Crans D.C., Grincienė G. (2009). Deprotonation of β-cyclodextrin in alkaline solutions. Carbohydr. Res..

[20] Loftsson T., Brewster Marcus E. (2012). Cyclodextrins as functional excipients: Methods to enhance complexation efficiency. J. Pharm. Sci..

[21] Saha S., Roy A., Roy K., Roy M.N. (2016). Study to explore the mechanism to form inclusion complexes of β-cyclodextrin with vitamin molecules. Sci. Rep..

[22] Coleman A.W., Nicolis I., Keller N., Dalbiez J.P. (1992). Aggregation of cyclodextrins: An explanation of the abnormal solubility of β-cyclodextrin. J. Incl. Phenom. Mol. Recognit. Chem..

[23] Sabadini E., Cosgrove T., Egídio F.D.C. (2006). Solubility of cyclomaltooligosaccharides (cyclodextrins) in H_2_O and D_2_O: A comparative study. Carbohydr. Res..

[24] Naidoo K.J., Chen J.Y.-J., Jansson J.L.M., Widmalm G., Maliniak A. (2004). Molecular properties related to the anomalous solubility of β-cyclodextrin. J. Phys. Chem. B.

[25] Cai W., Sun T., Shao X., Chipot C. (2008). Can the anomalous aqueous solubility of β-cyclodextrin be explained by its hydration free energy alone?. Phys. Chem. Chem. Phys..

[26] Duchěne D., Wouessidjewe D. (1990). Pharmaceutical uses of cyclodextrins and derivatives. Drug Dev. Ind. Pharm..

[27] Szejtli J. (1984). Highly soluble β-cyclodextrin derivatives. Starch-Stärke.

[28] Loftsson T., Duchêne D. (2007). Cyclodextrins and their pharmaceutical applications. Int. J. Pharm..

[29] Davis M.E., Brewster M.E. (2004). Cyclodextrin-based pharmaceutics: Past, present and future. Nat. Rev. Drug Discov..

[30] Wenz G. (2012). Influence of intramolecular hydrogen bonds on the binding potential of methylated β-cyclodextrin derivatives. Beilstein J. Org. Chem..

[31] Miranda J.C.D., Martins T.E.A., Veiga F., Ferraz H.G. (2011). Cyclodextrins and ternary complexes: Technology to improve solubility of poorly soluble drugs. Braz. J. Pharm. Sci..

[32] Hanna K., de Brauer C., Germain P. (2004). Cyclodextrin-enhanced solubilization of pentachlorophenol in water. J. Environ. Manag..

[33] Tavornvipas S., Arima H., Hirayama F., Uekama K., Ishiguro T., Oka M., Hamayasu K., Hashimoto H. (2002). Some pharmaceutical properties of a new branched cyclodextrin, 6-O-α-(4-*O*-α-d-Glucuronyl)-d-glucosylβ-cyclodextrin. J. Incl. Phenom. Macrocycl. Chem..

[34] Hirayama F., Mieda S., Miyamoto Y., Arima H., Uekama K. (1999). Heptakis(2,6-di-*O*-methyl-3-*O*-acetyl)-β-cyclodextrin: A water-soluble cyclodextrin derivative with low hemolytic activity. J. Pharm. Sci..

[35] Tongiani S., Ozeki T., Stella V.J. (2009). Sulfobutyl ether-alkyl ether mixed cyclodextrin derivatives with enhanced inclusion ability. J. Pharm. Sci..

[36] Fenyvesi É., Szemán J., Csabai K., Malanga M., Szente L. (2014). Methyl-beta-cyclodextrins: The role of number and types of substituents in solubilizing power. J. Pharm. Sci..

[37] Del Valle E.M.M. (2004). Cyclodextrins and their uses: A review. Process Biochem..

[38] Yong C.W., Washington C., Smith W. (2008). Structural behaviour of 2-hydroxypropyl-β-cyclodextrin in water: Molecular dynamics simulation studies. Pharm. Res..

[39] Terekhova I.V., Kumeev R.S., Al’per G.A. (2007). The interaction of caffeine with substituted cyclodextrins in water. Russ. J. Phys. Chem. A.

[40] Müller B.W., Brauns U. (1986). Hydroxypropyl-β cyclodextrin derivatives: Influence of average degree of substitution on complexing ability and surface activity. J. Pharm. Sci..

[41] Leclercq L., Bricout H., Tilloy S., Monflier E. (2007). Biphasic aqueous organometallic catalysis promoted by cyclodextrins: Can surface tension measurements explain the efficiency of chemically modified cyclodextrins?. J. Colloid Interface Sci..

[42] Loftsson T., Jarho P., Másson M., Järvinen T. (2005). Cyclodextrins in drug delivery. Expert Opin. Drug Deliv..

[43] Szente L., Fenyvesi É. (2017). Cyclodextrin-lipid complexes: Cavity size matters. Struct. Chem..

[44] Legrand F.-X., Sauthier M., Flahaut C., Hachani J., Elfakir C., Fourmentin S., Tilloy S., Monflier E. (2009). Aqueous hydroformylation reaction mediated by randomly methylated β-cyclodextrin: How substitution degree influences catalytic activity and selectivity. J. Mol. Catal. A Chem..

[45] Azarbayjani A.F., Lin H., Yap C.W., Chan Y.W., Chan S.Y. (2010). Surface tension and wettability in transdermal delivery: A study on the in-vitro permeation of haloperidol with cyclodextrin across human epidermis. J. Pharm. Pharmacol..

[46] Kiss T., Fenyvesi F., Bácskay I., Váradi J., Fenyvesi É., Iványi R., Szente L., Tósaki Á., Vecsernyés M. (2010). Evaluation of the cytotoxicity of β-cyclodextrin derivatives: Evidence for the role of cholesterol extraction. Eur. J. Pharm. Sci..

[47] Armstrong D.W.R., Faulkner J., Han S.M. (1988). Use of hydroxypropyl- and hydroxyethyl-derivatized β-cyclodextrins for the thin-layer chromatographic separation of enantiomers and diastereomers. J. Chromatogr. A.

[48] Francotte E., Brandel L., Jung M. (1997). Influence of the degree of substitution of cyclodextrin sulfobutyl ether derivatives on enantioselective separations by electrokinetic chromatography. J. Chromatogr. A.

[49] Okada Y., Matsuda K., Hara K., Hamayasu K., Hashimoto H., Koizumi K. (1999). Properties and the inclusion behavior of 6-*O*-α-d-Galactosyl- and 6-*O*-α-d-Mannosyl-cyclodextrins. Chem. Pharm. Bull..

[50] Higuchi T. (1960). Physical chemical analysis of percutaneous absorption process from creams and ointments. J. Soc. Cosmet. Chem..

[51] Schaefer H., Schalla W., Zesch A., Stüttgen G. (1982). Skin Permeability.

[52] Gallaher D., Plank D. (2015). α-Cyclodextrin as a food ingredient to reduce fat absorption. Agro Food Ind. Hi-Tech.

[53] Jambhekar S.S., Breen P. (2016). Cyclodextrins in pharmaceutical formulations I: Structure and physicochemical properties, formation of complexes, and types of complex. Drug Discov. Today.

[54] Loftsson T., Magnúsdóttir A., Másson M., Sigurjónsdóttir J.F. (2002). Self-association and cyclodextrin solubilization of drugs. J. Pharm. Sci..

[55] Wang C., Wang X., Xu X., Liu B., Xu X., Sun L., Li H., Zhang J. (2016). Simultaneous high-throughput determination of interaction kinetics for drugs and cyclodextrins by high performance affinity chromatography with mass spectrometry detection. Anal. Chim. Acta.

[56] Li H., Ge J., Guo T., Yang S., He Z., York P., Sun L., Xu X., Zhang J. (2013). Determination of the kinetic rate constant of cyclodextrin supramolecular systems by high performance affinity chromatography. J. Chromatogr. A.

[57] Wang C., Ge J., Zhang J., Guo T., Chi L., He Z., Xu X., York P., Sun L., Li H. (2014). Multianalyte determination of the kinetic rate constants of drug–cyclodextrin supermolecules by high performance affinity chromatography. J. Chromatogr. A.

[58] Jansook P., Moya-Ortega M.D., Loftsson T. (2010). Effect of self-aggregation of γ-cyclodextrin on drug solubilization. J. Incl. Phenom. Macrocycl. Chem..

[59] Saokham P., Loftsson T. (2015). A new approach for quantitative determination of γ-cyclodextrin in aqueous solutions: Application in aggregate determinations and solubility in hydrocortisone/γ-cyclodextrin inclusion complex. J. Pharm. Sci..

[60] Saokham P., Do T.T., Van den Mooter G., Loftsson T. (2018). Inclusion complexes of p-hydroxybenzoic acid esters and γ-cyclodextrin. J. Incl. Phenom. Macrocycl. Chem..

[61] Schönbeck C., Madsen T.L., Peters G.H., Holm R., Loftsson T. (2017). Soluble 1:1 complexes and insoluble 3:2 complexes—Understanding the phase-solubility diagram of hydrocortisone and γ-cyclodextrin. Int. J. Pharm..

[62] Lehner S.J., Müller B.W., Seydel J.K. (1994). Effect of hydroxypropyl-β-cyclodextrin on the antimicrobial action of preservatives. J. Pharm. Pharmacol..

[63] Loftsson T., Stefánsdóttir Ó., Friôriksdóttir H., Guômundsson Ö. (1992). Interactions between preservatives and 2-hydroxypropyl-β-cyclodextrin. Drug Dev. Ind. Pharm..

[64] Holm R., Olesen N.E., Alexandersen S.D., Dahlgaard B.N., Westh P., Mu H. (2016). Thermodynamic investigation of the interaction between cyclodextrins and preservatives—Application and verification in a mathematical model to determine the needed preservative surplus in aqueous cyclodextrin formulations. Eur. J. Pharm. Sci..

[65] Loftsson T. (1998). Increasing the cyclodextrin complexation of drugs and drug biovailability through addition of water-soluble polymers. Pharmazie.

[66] Jansook P., Kurkov S.V., Loftsson T. (2010). Cyclodextrins as solubilizers: Formation of complex aggregates. J. Pharm. Sci..

[67] Messner M., Kurkov S.V., Jansook P., Loftsson T. (2010). Self-assembled cyclodextrin aggregates and nanoparticles. Int. J. Pharm..

[68] Loftsson T., Hreinsdóttir D., Másson M. (2005). Evaluation of cyclodextrin solubilization of drugs. Int. J. Pharm..

[69] Savolainen J., Järvinen K., Taipale H., Jarho P., Loftsson T., Järvinen T. Coadministration of a water-soluble polymer increases the usefulness of cyclodextrins in solid oral dosage forms. Proceedings of the Ninth International Symposium on Cyclodextrins.

[70] Jansook P., Loftsson T. (2009). CDs as solubilizers: Effects of excipients and competing drugs. Int. J. Pharm..

[71] Kim Y., Oksanen D.A., Massefski J.W., Blake J.F., Duffy E.M., Chrunyk B. (1998). Inclusion complexation of ziprasidone mesylate with β-cyclodextrin sulfobutyl ether. J. Pharm. Sci..

[72] Omari M.M.A., Zughul M.B., Davies J.E.D., Badwan A.A. (2006). Factors contributing to solubility synergism of some basic drugs with β-cyclodextrin in ternary molecular complexes. J. Incl. Phenom. Macrocycl. Chem..

[73] Loftsson T., Matthíasson K., Másson M. (2003). The effects of organic salts on the cyclodextrin solubilization of drugs. Int. J. Pharm..

[74] He Y., Li P., Yalkowsky S.H. (2003). Solubilization of fluasterone in cosolvent/cyclodextrin combinations. Int. J. Pharm..

[75] Soltani N., Shaynafar A., Djozan D., Jouyban A. (2013). Solubility of three basic drugs in propylene glycol + water mixtures in the presence of β-cyclodextrin. J. Drug Deliv. Sci. Technol..

[76] Müller B.W., Albers E. (1991). Effect of hydrotropic substances on the complexation of sparingly soluble drugs with cyclodextrin dervatives and the influence of cyclodextrin complexation on the pharmacokinetics of the drugs. J. Pharm. Sci..

[77] Loftsson T., Sigurðardóttir A.M. (1994). The effect of polyvinylpyrrolidone and hydroxypropyl methylcellulose on HPβCD complexation of hydrocortisone and its permeability through hairless mouse skin. Eur. J. Pharm. Sci..

[78] Loftsson T., Frikdriksdóttir H., Sigurkdardóttir A.M., Ueda H. (1994). The effect of water-soluble polymers on drug-cyclodextrin complexation. Int. J. Pharm..

[79] Loftsson T., Másson M. (2004). The effects of water-soluble polymers on cyclodextrins and cyclodextrin solubilization of drugs. J. Drug Deliv. Sci. Technol..

[80] Yamakawa T., Nishimura S. (2003). Liquid formulation of a novel non-fluorinated topical quinolone, T-3912, utilizing the synergic solubilizing effect of the combined use of magnesium ions and hydroxypropyl-β-cyclodextrin. J. Control. Release.

